# Linking Geology and Microbiology: Inactive Pockmarks Affect Sediment Microbial Community Structure

**DOI:** 10.1371/journal.pone.0085990

**Published:** 2014-01-24

**Authors:** Thomas H. A. Haverkamp, Øyvind Hammer, Kjetill S. Jakobsen

**Affiliations:** 1 Centre for Ecological and Evolutionary Synthesis, Department of Biosciences, University of Oslo, Oslo, Norway; 2 Natural History Museum, University of Oslo, Oslo, Norway; 3 Microbial Evolution Research Group, Department of Biosciences, University of Oslo, Oslo, Norway; Wageningen University, Netherlands

## Abstract

Pockmarks are geological features that are found on the bottom of lakes and oceans all over the globe. Some are active, seeping oil or methane, while others are inactive. Active pockmarks are well studied since they harbor specialized microbial communities that proliferate on the seeping compounds. Such communities are not found in inactive pockmarks. Interestingly, inactive pockmarks are known to have different macrofaunal communities compared to the surrounding sediments. It is undetermined what the microbial composition of inactive pockmarks is and if it shows a similar pattern as the macrofauna. The Norwegian Oslofjord contains many inactive pockmarks and they are well suited to study the influence of these geological features on the microbial community in the sediment. Here we present a detailed analysis of the microbial communities found in three inactive pockmarks and two control samples at two core depth intervals. The communities were analyzed using high-throughput amplicon sequencing of the 16S rRNA V3 region. Microbial communities of surface pockmark sediments were indistinguishable from communities found in the surrounding seabed. In contrast, pockmark communities at 40 cm sediment depth had a significantly different community structure from normal sediments at the same depth. Statistical analysis of chemical variables indicated significant differences in the concentrations of total carbon and non-particulate organic carbon between 40 cm pockmarks and reference sample sediments. We discuss these results in comparison with the taxonomic classification of the OTUs identified in our samples. Our results indicate that microbial communities at the sediment surface are affected by the water column, while the deeper (40 cm) sediment communities are affected by local conditions within the sediment.

## Introduction

Pockmarks are craterlike structures found on the seabed [1]. They can be found in all oceans and even in lakes and can be very numerous in certain areas [2], [3]. They are often associated with subsurface oil and gas fields which makes them interesting geological features for the oil/gas-industry [4]. Pockmarks are often formed due to active processes in the subsurface such as the emission of gas and/or fluids to the surface. The exact formation of pockmarks is still under debate, but recent studies indicate that pockmark craters are formed rapidly, when pressurized subsurface gas or pore-water is suddenly released through the seafloor sediments [5], [6]. Following the sudden “birth” of pockmarks, many of these structures continue to emit gas or fluid from the subsurface at a slower pace until they become dormant after a relatively short active period [2], [3], [6]. During the expulsion of fluids and gas fine grained sediments are resuspended in the water column and deposited outside the pockmarks leaving coarser grain sized material inside the pockmark [6]. Dormant or inactive pockmarks can be awakened by new pulses of gas or fluid, indicated by the vertical stacking in the subsurface [3]. Areas with many pockmarks are often stable in the number of pockmarks since subsurface gas or fluid flow usually tends to follow the existing venting channels instead of creating novel ones [5]. Finally, surveys of the seabed indicate that inactive pockmarks outnumber the active pockmarks [2], [7].

Although inactive pockmarks may seem unexciting compared to active pockmarks, there are a number of studies describing the geological characteristics of these structures at different geographical locations [7]–[11]. For instance, since inactive pockmarks have no active outflow of gas and fluids it is expected that they would fill up over time due to sedimentation of particles. However, studies of inactive pockmarks in the Oslofjord and the Belfast Bay contradict such expectations. This suggests that some kind of activity keeps them open, or that they have been active up to recently [7], [9], [10]. A possible explanation is that pockmarks influence the hydrodynamics above the seabed. Pockmarks can have an effect on the local hydrodynamic conditions by deflecting the water current [12], [13]. The resulting upwelling of seawater could reduce the sedimentation rates of fine-grained particles inside the pockmarks, which would prevent the pockmarks from filling up. In a recent study in the Oslofjord a single inactive pockmark was intensively investigated to understand the reduced sedimentation rates within such structures [14]. It was shown that sediment traps placed closely above the seafloor had higher sedimentation rates inside the pockmark than outside the pockmark. Nonetheless, the pockmark sediments contained relatively larger abundances of the coarser particles compared to the surrounding sediments. This suggested that a large fraction of the fine-grained particles are resuspended inside pockmarks due to turbulence and possible biological activity. The resuspended particles could then be transported out of the pockmarks by water currents [14]. In this way, inactive pockmarks can be maintained via physically or biologically induced water movements.

Pockmarks are not only geologically interesting structures but are of biological significance as well. For instance active pockmarks, so called because of detectable gas and fluid fluxes, harbor specialized microbial communities metabolizing compounds such as methane or other hydrocarbons [15]–[17]. These microbial communities provide energy and nutrients to sustain the presence of specialized macroorganisms living in symbiotic interactions with the microbes. Due to the exotic nature of these chemoautotrophic communities they have been intensively studied in recent years [18].

In contrast to active pockmarks their inactive counterparts have been studied to a limited extent in a microbiological perspective and then only in relation to the presence of biogenic methane or remains of hydrocarbon seepage [19]–[21]. Additionally, to the best of our knowledge we are not aware of studies comparing microbial communities of inactive pockmarks and the surrounding sediments that are not influenced by hydrocarbons. For macrofauna on the other hand, there is literature available describing communities inside and outside pockmarks. In brief, bioturbating macrofaunal species in sediment communities were found to be significantly different inside and outside of pockmarks at several locations in the Oslofjord suggesting that pockmarks influence the distribution of macrofaunal species [11]. Bioturbators can both redistribute particles and/or ventilate the sediments by moving water in or out of the burrows, which could affect both redox gradients and availability of microbial resources (e.g. carbon, nitrogen) [22]. Therefore changes in bioturbating species composition could have an effect on the sediment biogeochemistry, which in turn can influence the microbial community composition inside and outside pockmarks [22]–[29].

Other factors influencing the biogeochemistry of benthic sediments are sinking phytoplankton blooms, marine snow and zooplankton fecal pellets and terrestrial run off that add organic matter to the sediments [30], [31]. Organic matter, which is part of total organic carbon (TOC), is made up of simple and complex compounds such as sugars, proteins, lipids, humic acids, etc., and contributes to the total carbon (TC) content of the sediments. In addition, sediment TC also contains inorganic carbon (IC) compounds such as carbonate, bicarbonate and dissolved carbon dioxide. The microbial sediment community decomposes the organic matter and releases dissolved organic carbon (DOC), which can be used by other members of the microbial community. As pelagic organic matter is deposited on the seabed, it can induce rapid changes within the sediments that can affect the diversity of the benthic microbial communities [32], [33]. Bienhold et al. [33] showed that an increase of organic matter (phytodetritus) deposition onto sediments increased the bacterial operational taxonomic unit (OTU) richness in oligothrophic conditions. However, this effect was not seen under mesotrophic conditions suggesting that other factors e.g. oxygen availability, become limiting.

Besides organic matter an additional carbon source is included in TOC which is composed of polycyclic aromatic hydrocarbons (PAHs) derived from natural or anthropogenic sources [34], [35]. Due to their hydrophobic nature most PAHs will absorb to sediment particles from the water column where they will be available for biodegradation [34], [36]. PAHs vary in their structure and molecular weight where an increase in molecular weight enhances the degradation and environmental persistence times of these compounds [35], [36]. This means that not only the PAH sediment concentrations decrease with depth due to biodegradation, but that the ratios between different PAHs also change with time. In addition, several studies have used the PAH ratios to determine historic anthropogenic PAH input into marine and freshwater sediments [37]–[39]. The rate at which biodegradation of PAHs occurs depends on many factors including temperature, pH, oxygen availability, the microbial community composition, etc., [35]. The key players in the biodegradation of PAH are bacteria and lignolytic fungi, which mineralize it into inorganic minerals and inorganic carbon released as DOC.

Bacteria are not only important for the carbon cycling in the sediment, but are also major players in the nitrogen cycle [40]. The nitrogen found in sediments can be composed of nitrate, nitrite, ammonia and organic nitrogen sources such as amino acids. Bioavailable nitrogen can come from degradation of organic matter or via nitrogen fixation [31]. In the presence of oxygen ammonium is used in nitrification giving nitrate while in anoxic sediments ammonium is converted to N_2_ via the anammox pathway [41]. Nitrate is metabolized in anoxic sediments via denitrification or dissimilatory nitrate reduction to ammonium. Organic matter content of marine sediments determines the denitrification rates since nitrate is an electron acceptor for the oxidation of organic matter, which is considered an important process in coastal marine sediments [42], [43].

Considering that inactive pockmarks in coastal zones are influenced in the same way as normal sediments then the main driver for the microbial sediment community diversity is the organic loading from the water column through sedimentation. This implies that pockmark sediment communities would be dominated by *Delta*- and *Gammaproteobacteria* as is the case for normal marine sediments [44]. However, a few studies suggest that sedimentation rates within pockmarks could be different from outside and it is not clear how this affects both the deposition of (in-) organic matter and the microbial community composition inside and outside of pockmarks [12]–[14], [45], [46].

Since studies of microbial communities from hydrocarbon negative inactive pockmarks are lacking, we have compared these communities with surrounding sediments in the Oslofjord using amplicon sequencing of the 16S rRNA. The Oslofjord pockmarks are relatively recently formed and do not seem to be influenced by hydrocarbons [10]. Analysis of two cores from the Oslofjord indicated that these pockmarks originate from the start of the Holocene and that they are influenced by seepage of fresh groundwater, but that the seepage is sporadic [9]. It is therefore not likely that seepage counters sedimentation rates between seepage events. Nonetheless, if seepage occurs it could affect ion concentrations of Na^+^, Cl^−^ and SO_4_^2+^ within the sediments [47]. In line with this, freshwater seepage could in principle have an effect on the microbial communities via changes in the redox potential of the sediments with the deeper lying communities being more easily affected [47], [48]. Furthermore, it is well known that the redox potential in sediments changes with depth and in recent years it was shown that this affects the cell abundances as well as the microbial community composition [31], [49]–[52].

Here we have tested the hypothesis that the inactive Oslofjord pockmarks have a different microbial community than the surrounding sediments caused by processes within the pockmark structures. Since it is unclear which processes exactly determine the divergence of the microbial communities within pockmark sediments from the surrounding sediments, it was needed to obtain a detailed description of the chemistry and community composition. This would allow us to identify major factors causing a community difference between inactive pockmarks and their surrounding sediments.

## Materials and Methods

### Ethics Statement

No specific permits were required for the described study. The study location is not privately owned or protected in any way and the sediment sampling did not involve endangered or protected species.

### Sample Collection

**Figure 1:**
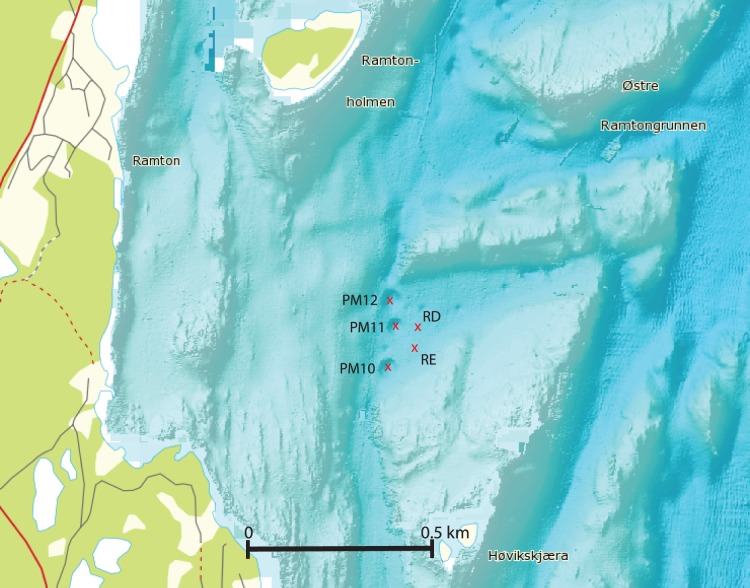
Bathymetric map of the sampling area in the Oslofjord. The red crosses indicate the sampling sites and the sampling site designation is given. The map was generated with the www.mareano.no website.

Sampling of Oslofjord sediments was done on the 28^th^ of October 2011, using the research vessel *Trygve Braarud*. Among the more than 500 Oslofjord pockmarks we chose three pockmarks (PM) that are located in the inner Oslofjord at a depth of about 70 meters and two closely related reference sites (RD and RE) for sampling (59° 44′ N, 10° 31′ E; Table 1, Figure 1). The maximum distance between sampling sites was 180 m (PM10 vs. PM12) (Table 1, Table S1 in File S1). Using a multicorer, we obtained three sediment cores from every sample site that were used for DNA extraction and chemical analysis. The cores (diameter × length: 10×60 cm) were transported to the laboratory and the cores were pushed out and divided into two halves along the vertical axis using a platinum wire. Visual observations of the sediment coloration were noted (Table S2 in File S1). One half of the core was used for extracting porewater, while the other half was used for collecting material for DNA extraction and PAH measurements. We sampled the cores at the horizons of 0–4 and 40 cm depth for three reasons. First, sediments are known to have a highly structured separation of different microbial communities along the depth axis [53], [54]. Second, the Oslofjord pockmarks show indications of sporadic freshwater seepage below 30 cm depth [9].Third, the pockmark communities in the Oslofjord experience both higher sedimentation and resuspension rates which could affect the microbial community composition within the pockmark sediments [13], [14]. All extractions were done using three cores on the 0–4 cm and 40 cm sediment horizons, giving a total of 30 samples. Sediment samples for DNA extraction were frozen at −20°C. Porewater was extracted using a Rhizon CSS-F 5 cm sampler (Rhizosphere Research Products, The Netherlands). The extracted porewater was divided into two batches and immediately frozen after extraction and stored at −20°C until measurement. For PAH measurements, >20 gram of sediment was collected from one core per location and stored in dark brown 100 ml glass bottles, frozen at −20°C.

**Table 1:** Description of Oslofjord sampling sites.

Geology	Sample ID	Latitude	Longitude	Pockmark diameter(m)	Pockmark depth(m)	Water depth surroundingseabed (m)	Average distance to other sample sites (m)
Pockmark	PM10	59 44.168 N	10 31.538 E	44.7	7.7	65.9	108
Pockmark	PM11	59 44.231 N	10 31.551 E	33.9	6.7	69.5	71
Pockmark	PM12	59 44.265 N	10 31.531 E	48.7	7.9	68.7	103
normal seabed	RD	59 44.233 N	10 31.635 E	na	na	69.0	83
normal seabed	RE	59 44.196 N	10 31.621 E	na	na	66.6	82

* Diameter and depth of the pockmarks was determined via the methods described in Webb et al., 2009.

# Distances were calculated based on the geographical coordinates. Distances between all sites can be found in Table S1 in File S1.

### Chemical Analysis

One batch of sediment porewater was used to measure concentrations of the cations: Na^+^, K^+^, Mg^2+^, Ca^2+^ and anions: F^−^,Cl^−^, SO_4_^2−^, Br^−^. The other batch was used for total nitrogen (TN), non-purgable organic carbon (NPOC) and IC (Table S3 in File S1). For the ions measurements, 1 ml of porewater was diluted 100x and 1000x. The cations were measured on an Ion Chromatography System (ICS-1000) and the anions on an Ion Chromatography System (ICS-2000) with the appropriate standards (Thermo Scientific, CA, USA).

Total Nitrogen, NPOC and IC concentrations were measured on a Thermo Finnigan Flash 1112 element analyzer (Interscience, The Netherlands). PAHs, TC and TOC measures were performed by Geolab Nor (Norway) on dried and ground sediments. Detailed descriptions of the sample sites, cores and chemistry data can be obtained from the website: http://doi.pangaea.de/10.1594/PANGAEA.820086 (File S1).

### DNA Extraction and Clean-up

Sediment samples were thawed at 4°C and four replicate samples were taken for each core and each depth. DNA extraction was done using the FastDNA spin kit for soil (MP biomedicals, OH, USA) following the manufacturer’s instructions with minor changes. In brief, approximately 0.5 g of sediment was weighed and transferred to a bead-beating tube. Subsequently, NaPO_4_ buffer and MT buffer were added to the tube, and the tube was frozen at −20°C for at least 30 minutes. The samples were thawed and thereafter homogenized using the Fastprep instrument for 2 times 20 seconds at speed 6. Homogenized samples were centrifuged at 14.000×g in an Eppendorf centrifuge 5424R (Eppendorf AG, Hamburg, Germany) and the normal protocol was followed until elution. Elution was done by adding 100 µl of DNase-free water and samples were incubated at 55°C for 3 minutes. After incubation, samples were centrifuged at 14.000×g for one minute. The DNA extractions were stored at 4°C. DNA extractions of each replicate were checked by separating the products by electrophoresis in a 1% agarose gel (Seakem LE agarose, Lonza group ltd, Basel, Switzerland). The four replicates for each of the 30 samples were pooled. DNA concentrations were measured using a Nanodrop ND-1000 (Nanodrop Technologies, Wilmington, DE, USA), which indicated the presence of humic acids due to a large absorption peak between 220 and 230 nm.

To remove humic acids and other PCR inhibitors from the DNA extractions we used the Mobio Powerclean cleanup kit (Mobio Laboratories, Carlsbad, CA, USA) following the manufacturer’s instructions. Cleaned DNA preparations were checked using a 1% agarose gel. DNA concentration was measured using Nanodrop ND-1000.

### PCR Amplification, Normalization and Amplicon Sequencing

Amplification of the V3 region of the 16S rRNA was done using the primers 338F and 533R with the MID-tags and adaptors already attached [55] (File S2). For each sample triplicate 25 µl PCR reactions were set up with the following concentrations per reaction: 1×HF PCR Buffer (Thermo Scientific, Waltham, MA, USA), 10 ng DNA, 5 µM of each primer, 2 mM of each dTNP, and 0.5 units of Phusion Hot Start II DNA Polymerase (Thermo Scientific, Waltham, MA, USA). The PCR was run on a Biometra T1 Thermocycler with the following program: 60 s 98°C hotstart, followed by 20 cycles of 10 s at 98°C, 30 s at 55°C and 15 s at 72°C. The program finished with a 10 minutes elongation step at 72°C. The PCR reactions were accompanied by a positive (*Polaromonas naphthalenivorans* DSM15660) and a negative control (without added DNA). The PCR products were visualized on a 2% agarose gel (Seakem LE agarose, Lonza Group Ltd, Basel, Switzerland). PCR product concentration was normalized using the SequalPrep Normalization Plate (96) kit (Invitrogen, Paisley, UK), following the manufacturer’s instructions. 15 µl of each PCR reaction was bound to the normalization plate. Elution was performed with 20 µl of elution buffer. After elution all PCR reactions were pooled in two 2 ml Eppendorf tubes. The amplicon library was concentrated using the SV gel/PCR clean-up system (Promega, Fitchburg, WI, USA) following the provided instructions of the manufacturer. The entire library was loaded on one column. The library was eluted using 50 µl of DNase-free water with incubation for 60 seconds at 55°C. The elution step was repeated once. The eluted product was subsequently loaded on a 2% agarose gel and primer dimers were removed by excising the PCR band from the gel using a sterile razor. The gel with PCR product was subsequently dissolved using the SV gel/PCR clean-up system and eluted into 50 µl of DNase-free water. The final product was checked on a 2% agarose gel for the presence of primer dimers. The 16S rRNA V3 amplicon library was sequenced using ¾ of a plate (3 lanes) on a 454 GS-FLX sequencer at the Norwegian Sequencing Centre (Univ. of Oslo, Oslo, Norway) [56]. The raw sequences can be obtained from the SRA archive: accession number SRX264805.

### Quality Control

The Schloss SOP (www.mothur.org/wiki/Schloss_SOP) was followed to remove sequence noise and PCR artifacts in the sequence data with Mothur (version 1.29.1) [57]. In brief, the flowgrams in the sff file were separated into different samples using the MIDtags and forward primers. Sequences were trimmed to 320 flows and shorter sequences were discarded. Sequence noise was reduced using the shhh.flows command in Mothur. Subsequently, sequences with mismatches in forward primers and/or MID-tags were discarded. The reverse primers and MID-tags were removed after aligning the sequences against the Silva Bacterial reference alignment. In addition unalignable sequences were removed. Subsequently, preclustering was performed followed by chimera checking and removal using the UCHIME implementation in Mothur [58], [59]. Finally, we classified sequences against the RDP reference, to identify sequences classified as chloroplast, mitochondria, Archaea, or Eukarya, and these were subsequently removed. The final clean dataset used in the diversity analysis can be found in File S5.

### Diversity Analysis

Bacterial sequence diversity was analyzed for alpha and beta-diversity using Mothur [57]. Alpha-diversity is considered to be the diversity within one sample, while beta-diversity describes the difference in species composition, or turnover of species, among two or more samples [60].

To test the effect of removal of unique reads (singletons) on the alpha-diversity we discarded singleton sequences using the *split.abund* command in Mothur. In this way we created two datasets with or without unique reads. Both datasets were clustered at the 97% sequence similarity. Alpha-diversity was analyzed for both datasets. For alpha-diversity, sequence effort for every sample was taken into account using rarefaction curves, and diversity estimators were estimated based on standardized samples using the smallest sample (RDC40–2071 reads). Standardization was performed using the *sub.sample* command in Mothur [61]. The diversity estimators (Chao1, the non-parametric Shannon index, the inverse Simpson index and Good’s coverage) were calculated using 1000 bootstraps to determine confidence intervals (Table S4 in File S1) [61]. Rarefaction curves and rank-abundance curves were created using R-statistics (version 3.0.1). Venn diagrams to display the shared OTUs between the four sample groups were plotted using the R-package Venn Diagram version 1.6.0 and colored using RcolorBrewer version 1.0.

The remaining analysis used the data with unique reads removed. Taxonomic classification of OTUs was done using blast+ (version2.2.28) with the blastN algorithm against the SILVA V108 SSU database with standard settings except the maximum e-value was set to 1.0E-20 [62], [63]. The Blast output files for every sample were analyzed in MEGAN and compared using the following Lowest Common Ancestor Parameters: Minsupport: 1; Minscore: 155; Top-percent: 2% and the Percent Identity Filter activated [64].

To study if communities from the four sample groups were different we analyzed them using various qualitative beta-diversity indices. Mothur was used to calculate distances based on the Jaccard similarity index (community membership) and the Yue and Clayton Theta similarity coefficient (ThetaYC) (community structure) on the standardized OTU abundances. The calculated distances were used as input in Mothur to produce dendrograms based on hierarchical clustering with the unweighted pair group method with arithmetic mean (UPGMA) method. The generated dendrograms were analyzed using the parsimony test implemented in Mothur. To test for significant differences between the four sample groups in our analysis we used the parsimony test implemented in Mothur to analyse the different UPGMA dendrograms.

Using the Clearcut implementation in Mothur we created a phylogenetic tree to measure the genetic diversity between the communities using weighted and unweighted unifrac [65]. The unifrac distance matrices were used to perform ordination analysis with principal coordinates analysis (PCoA) in Mothur and visualized using R-statistics (version 3.0.1).

### Metastats Analysis

The Mothur implementation of Metastats was used to determine which OTUs showed a different abundance between the reference and pockmark samples at 40 cm depth [66]. To be significant we used a q-value (false discovery rate corrected p-value) of 0.01 as a maximum. We selected high abundance OTUs with a minimum mean abundance of 0.001 (calculated by Metastats) in at least one of the groups tested in order to increase reliability of the test. The fasta sequences of these OTUs were classified as discussed above.

### Statistical Analysis

We used the non-parametric Kruskal-Wallis test implemented in R-statistics to test if there were significant differences of the measured chemicals between 1) the two depths (0–4 cm vs 40 cm), 2) The sites (pockmarks vs. reference sites) and 3) between the four groups (PM04, R04, PM40, R40). A p-value <0.05 was considered significant. The hydrocarbons were not included. Due to the small and uneven sample size between the pockmarks and the sediments at both sediment depths we used an additional analysis with a two-sample permutation test implemented in the DAAG package (version 1.16) to test for significant differences for TC, TOC, NPOC and TN.

The package Vegan (version 2.0–8) was used for Analysis of Similarities (ANOSIM), ordination analysis and fitting of environmental parameters onto constrained correspondence analysis (CCA) ordinations. ANOSIM was performed using the Jaccard index, ThetaYC index, unweighted unifrac and weighted unifrac distance matrixes generated in Mothur with either depth or the four groups as variables and with 10000 permutations.

CCA was performed on the subsampled abundance table (Standardized on the smallest sample (RDC40–2071 reads)) to estimate the relation between community composition and the chemical parameters measured except the PAHs. Akaike’s information criterion (AIC) with forward selection was used to identify those chemical variables that explained most of the variation between the communities. TC was identified as the main determinant and was used to constrain the correspondence analysis. Statistical significance of the constrained correspondence analysis was tested using an ANOVA like permutation test with 1000 permutation using the function *anova.cca* (Vegan). The environmental variables (TC; TOC; IC; NPOC; TN; Na^+^; K^+^; Mg^2+^; Ca^2+^; F^−^; Cl^−^; SO_4_^2−^; Br^−^) were tested for significant correlation with the sample distribution in the CCA analysis using the *envfit* command, with 999 permutations (vegan). Only those factors were fitted that had a p-value <0.01.

## Results

### Different Sediment Geochemistry between Pockmark and Reference Sites at Two Depths

To address if the pockmark sediments (PM10, PM11 and PM12) are chemically different from the non-pockmark sediments (RD and RE) we measured 13 different variables in all samples and an additional 17 polycyclic aromatic hydrocarbons (PAHs) in only one core at each sample location (Table 2; Table S3 in File S1). Sediments are highly stratified ecosystems in regard to their depth profile, which should be apparent from our chemical data. We used the non-parametric Kruskal-Wallis test (KW-test) to identify which environmental variables had significantly different concentrations between the two depths in both pockmark and reference samples (Table 2). The concentrations of TC, TOC and TN (all three variables: p<0.001) were significantly different between 0–4 cm and 40 cm depth. The other variables did not show significant differences between the two depths. TN concentrations are 0.19 (+/−0.08) mg l^−1^ and 0.58 (0.09) mg l^−1^ in the surface and deep samples, respectively. The TC concentrations at 0–4 and 40 cm were 3.56 (+/−0.22) % C and 1.36 (+/−0.18) % C respectively, while the TOC concentrations were 2.41 (+/−0.14) % C and 0.92 (+/−0.21) % C for 0–4 and 40 cm respectively. In a similar way the total polycyclic aromatic hydrocarbons (ΣPAHs) concentrations were high in the 0–4 cm zone (1.81 mg kg^−1^ (+/−0.26) and low at 40 cm depth (0.08 mg kg^−1^ (+/−0.02) (KW-test: p<0.01). Interestingly, the DNA concentrations of our samples showed the same pattern. The 0–4 cm horizon (35.5±4.3 ng µl^−1^) has a higher DNA concentration than the 40 cm sediment horizon (6.4±1.3 ng µl^−1^) (KW-test: p<0.01).

**Table 2:** Overview of a selection of chemical variables measured per sample.

Sample ID	Sedimentdepth (cm)	TC(% dry weight)	TOC(% dry weight)	Na $(ppm)	F #(ppm)	NPOC(mg l-1)	TN(mg l-1)	IC(mg l-1)	Sum 16PAHs (µg/kg)
PM10A04	0	3.66	2.53	10178	71.3	2.235	0.209	0.478	1668
PM10B04	0	3.78	2.56	11225	70.8	2.285	0.308	0.414	nd
PM10C04	0	3.76	2.54	17747	68.3	2.470	0.327	0.469	nd
PM11A04	0	3.65	2.46	12643	71.0	2.426	0.331	0.440	2278
PM11B04	0	2.98	2.03	12752	70.3	1.342	0.185	0.459	nd
PM11C04	0	3.8	2.43	11291	71.0	0.971	0.122	0.507	nd
PM12A04	0	3.53	2.36	13621	72.6	1.234	0.152	0.254	1528
PM12B04	0	3.54	2.38	11052	57.4	1.216	0.196	0.560	nd
PM12C04	0	3.45	2.4	10458	71.4	1.127	0.170	0.307	nd
RDA04	0	3.45	2.4	11024	70.9	1.599	0.139	0.430	1860
RDB04	0	3.73	2.28	12260	66.9	1.257	0.117	0.509	nd
RDC04	0	3.4	2.35	13810	70.6	1.472	0.111	0.526	nd
REA04	0	3.3	2.31	11266	71.1	0.969	0.177	0.490	1735
REB04	0	3.59	2.53	10078	72.2	1.412	0.234	0.339	nd
REC04	0	3.77	2.54	10210	16.9	0.766	0.120	0.302	nd
PM10A40	40	1.29	0.84	10469	71.1	2.104	0.654	0.441	67
PM10B40	40	1.24	0.82	9995	71.1	2.994	0.776	0.365	nd
PM10C40	40	1.48	1.03	9917	71.4	2.247	0.659	0.331	nd
PM11A40	40	1.23	0.81	12664	70.4	2.203	0.713	0.497	54
PM11B40	40	1.07	0.55	13289	69.7	2.071	0.482	0.533	nd
PM11C40	40	1.07	0.58	10746	70.5	2.537	0.518	0.217	nd
PM12A40	40	1.36	0.83	11891	70.6	1.275	0.501	0.262	79
PM12B40	40	1.23	0.84	13299	69.7	1.467	0.564	0.575	nd
PM12C40	40	1.24	0.74	15667	69.4	1.467	0.556	0.072	nd
RDA40	40	1.63	1.19	13973	70.0	1.430	0.503	0.586	117
RDB40	40	1.45	1.07	10848	71.6	1.414	0.591	0.467	nd
RDC40	40	1.41	1.09	12067	41.2	1.335	0.602	0.307	nd
REA40	40	1.51	1.1	10091	71.9	1.178	0.557	0.560	79
REB40	40	1.55	1.16	12318	71.1	1.030	0.466	0.249	nd
REC40	40	1.58	1.15	9804	12.3	0.956	0.623	0.619	nd

$ The value here is the average of the back calculation for both measurements.

# Flouride ions were only measured with a 100x dilution. So it is 1 single measurement.

* nd: not determined.

In addition to sediment depth as a factor, we included the origin of the samples, e.g. pockmarks or reference samples as well in our analysis, which resulted in the following four groups: Reference 0–4 cm (R04), Reference 40 cm (R40), Pockmark 0–4 cm (PM04) and Pockmark 40 cm (PM40).

Testing the chemical variables for the combined effect of sediment depth and pockmark vs. reference sediments with the KW-test indicated that TC, TOC, TN and NPOC were significantly different between the four groups of samples (p-values are: <0.001; <0.001; <0.001; p = 0.03) (Table S3 in File S1). Comparing the concentrations at the 40 cm horizon indicated a significant difference between pockmarks and normal sediments for TC, TOC and NPOC (KW-test: p<0.01, while there was no such difference for TN (KW-test: p>0.05). The concentrations were lower at 40 cm depth in the pockmark sediments for TC (1.25% C +/−0.12 vs. 1.52% C +/−0.08) and TOC (0.78% C +/−0.14 vs. 1.12% C +/−0.04) compared to the reference samples. In contrast NPOC concentrations were higher in the 40 cm pockmark sediments compared to the reference samples at the same depth (2.04 mg l^−1^+/−0.55 vs. 1.22 mg l^−1^+/−0.22). For TN we did not find a significant difference at 40 cm depth (0.60 mg l^−1^+/−0.10 vs. 0.56 mg l^−1^+/−0.06). We confirmed the differences between the pockmarks and the reference sites at 40 cm depth using a two-sample permutation t-test showing similar results (data not shown).

In contrast to the concentrations at 40 cm, we found that at the 0–4 cm horizon TN concentrations were significantly different (KW-test: p = 0.045) (0.22 mg l^−1^+/−0.08 vs. 0.15 mg l^−1^+/−0.05). This could however not be not be confirmed by a non-parametric two-sample t-test with permutation (p = 0.07) (Table S3 in File S1).

The ratio between the concentrations of different PAHs (ANT/178, FLT/202, BAA/228 and IND/276) in the surface sediments all suggest that the origin of the PAHs comes from combustion sources (Table S3 in File S1) [38] which is expected in the anthropogenically influenced Oslofjord. The deeper sediments show lower levels for the ANT/178 ratio and the BAA/228, but are still suggesting combustion as the original source of the PAHs. The other two ratios did not show major changes although the IND/276 was slightly higher in the 40 cm sediment zone.

### Diversity Estimates

Microbial diversity of the pockmark sediments was studied using 454 pyrosequencing of the 16S rRNA V3 region. After denoising, removal of artifacts and chimeras a total of 228927 reads was obtained representing 33571 unique sequences. 19401 (57.8%) of the unique sequences were only represented by a single read (singletons), while 14170 sequences were represented by at least two reads. The high amount of singletons could be due to various errors such PCR base changes or chimera formation and this could lead to an over estimation of OTU numbers with deleterious effects on the alpha and beta-diversity estimations [67]–[70]. We therefore tested if the removal of singletons had a significant effect on the alpha diversity estimations after clustering the sequences at 97% sequence similarity. Removal of the singletons reduced the total dataset with ≈8.5% to 209526 reads (Table S2 in File S3). The total number of OTUs at the 97% cut-off dropped from 20473 to 9246 OTUs. This reduction of OTUs is less than the 19401 sequences removed, since a fraction of the singletons clustered using the 97% sequence cut-off when singletons were not removed.

**Figure 2:**
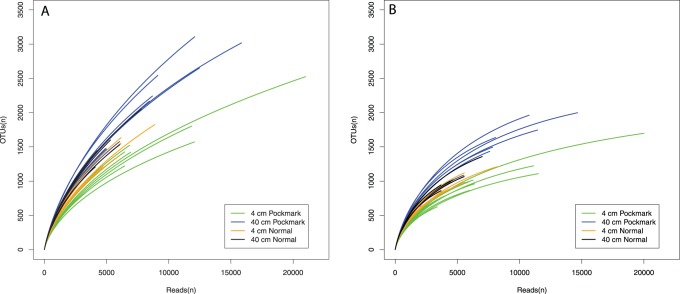
Rarefaction curves of 16S rRNA sequences at the 97% sequence similarity cut-off. A) Rarefaction curves with singletons included. B) Rarefaction curves with singletons excluded. The sample coloration descriptions are indicated in the figure. Samples are grouped color wise based on location (pockmark vs. reference sediments) and depth (4 cm vs. 40 cm).

Rarefaction analysis indicated a high diversity among the amplicons with the 40 cm horizon being the most diverse in both treatments of the data (Figure 2). Nonetheless, the rarefaction curves after removal of singletons show less diversity and the curves are less steep suggesting that we have captured more of the diversity in the sediments. This is also suggested by a slightly higher average Good’s coverage for all samples (average: 0.77 vs. 0.82) (Table 3).

**Table 3:** Diversity estimators for the Oslofjord sediment samples after removal of unique sequences.

Sample	Depth(cm)	Sequencecount	OTUs^97$^	SingletonOTUs^97^	StandardizedOTUs^97^ *	Chao1	NP Shannon#	Simpson1/D	Good's coverage
PM10A04	4	6013	866	335	521	946	5.25	35.96	0.86
PM10B04	4	11514	1109	366	576	1217	5.29	48.41	0.83
PM10C04	4	19997	1698	526	552	1054	5.46	33.20	0.85
PM11A04	4	11135	1224	429	569	994	5.40	57.75	0.85
PM11B04	4	6379	961	374	585	1110	5.32	59.18	0.84
PM11C04	4	3381	625	269	692	1271	5.32	99.61	0.81
PM12A04	4	3978	784	338	612	1089	5.56	65.34	0.84
PM12B04	4	6267	1015	364	611	1221	5.70	68.95	0.83
PM12C04	4	5957	993	414	741	1497	5.57	120.13	0.79
RDA04	4	4366	886	366	690	1222	5.81	136.33	0.82
RDB04	4	5561	1120	445	796	1636	5.99	150.10	0.77
RDC04	4	5628	984	404	781	1527	5.68	176.90	0.78
REA04	4	4177	869	383	692	1209	5.69	61.72	0.82
REB04	4	5616	1077	452	644	931	5.82	91.43	0.86
REC04	4	8170	1210	467	667	1189	5.68	66.12	0.82
PM10A40	40	4395	999	408	500	967	6.11	45.60	0.87
PM10B40	40	7596	1434	552	542	1086	6.12	48.69	0.85
PM10C40	40	10773	1962	675	497	814	6.34	45.28	0.88
PM11A40	40	5494	1086	413	604	1098	6.08	75.92	0.84
PM11B40	40	8075	1636	569	619	1080	6.46	90.74	0.84
PM11C40	40	14664	1998	566	602	1107	6.29	74.50	0.84
PM12A40	40	4464	991	409	651	1244	6.04	76.25	0.82
PM12B40	40	11449	1746	589	698	1211	6.30	138.97	0.82
PM12C40	40	7840	1496	546	846	1820	6.28	121.66	0.74
RDA40	40	4658	1019	396	843	1637	5.94	229.91	0.76
RDB40	40	3682	944	413	690	1185	6.08	102.45	0.82
RDC40	40	2071	644	293	780	1516	6.00	177.51	0.78
REA40	40	7000	1358	491	713	1207	6.11	82.33	0.82
REB40	40	5535	1065	410	750	1428	5.86	93.08	0.79
REC40	40	3691	860	335	663	1058	5.93	66.79	0.84

Diversity estimators are average values calculated on standardized counts based on the smallest sample with permutations (n = 1000). Standard deviations were omitted for clarity, but can be found in Table S4 in File S1.

$ OTUs^97^: operational taxonomic units at the 97% sequences similarity cut-off.

* Distance metrics were calculated after standardization of all samples to the smallest sample (RDC40 = 2071) and bootstrapped (n = 1000).

# Non-Parametric Shannon.

After removal of singletons only the non-parametric Shannon index showed a significant difference between the combined (pockmark and reference sites) surface (5.57+/−0.22) and the combined deep (6.13+/−0.17) samples (KW-test: p<0.01), while this was not the case for the other diversity estimators. In addition, the non-parametric Shannon index was significant when the four sample groups, depth (0–4 vs 40 cm) and location (pockmark vs. reference site) were used (KW-test: p<0.01). For the same test the Simpson 1/D index was as well significant (KW-test: p = 0.043), while the richness as measured with Chao1, was highly comparable between the surface and the deep samples when singletons are not included (KW-test: p>0.01) (Table 3, File S3).

The above results indicate that removal of singletons gives a more conservative analysis of the community differences and we therefore continue the discussion based on this notion.

**Figure 3:**
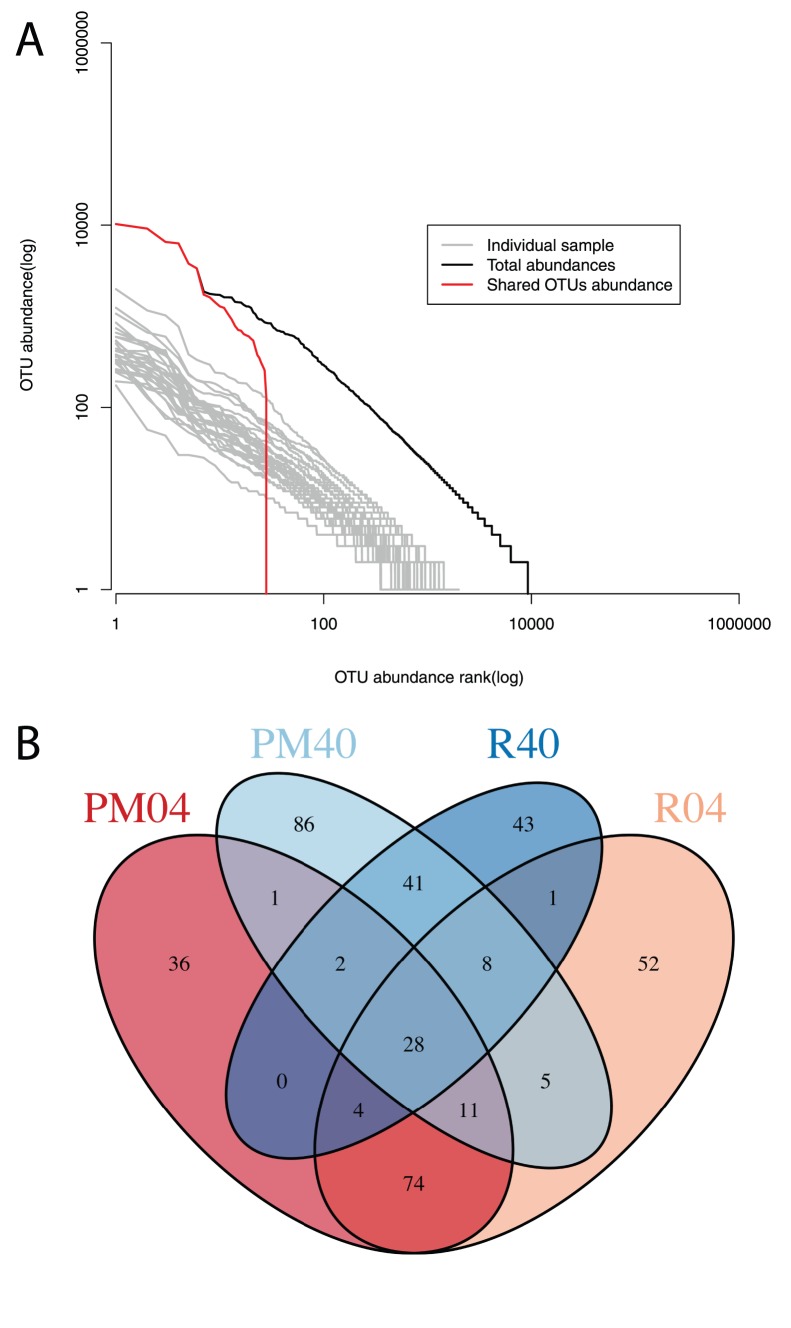
Shared OTUs between all samples. **A)** Rank abundance curves for all samples at the 97% sequence similarity cut-off. Black depicts the rank abundance curve for all OTUs in all samples. Red indicates the rank abundance curve for the 28 OTUs shared across all samples. In grey the rank abundance curves are plotted for the individual samples **B)** Venn diagram showing the number of OTUs shared between each of the four groups: Pockmark 0–4 cm (PM04), Pockmark 40 cm (PM40), Reference site 0–4 cm (R04) and Reference site 40 cm (R40).

Using rank-abundance curves we determined the distribution of reads over the different OTUs. All samples have fairly similar rank abundance curves (Figure 3A), with the major difference being due to sequencing effort. The rank abundance curve for all detected OTUs shows that one OTU has over 10000 sequences in our dataset. In addition, we find that 28 OTUs out of 9246 are shared among all samples with many of them belonging to the most abundant OTUs found in the dataset (Figure 3A & B). The number of shared OTUs between all samples per group was higher than 28 (PM04: 156; R04: 183; PM40: 182; R40: 127 OTUs). The number of OTUs shared between each of the groups is considerably lower with the least shared OTUs between the samples of groups R04 and R40 (41) (Figure 3B).

### Beta-diversity

Estimation of the beta-diversity in our experiment was done in several ways, using either various (dis-) similarity indices (measures) based on the OTU tables or phylogeny-based methods such as Unifrac. We did not test the effect of singleton removal on the beta-diversity estimations since various authors have shown that in most experimental set-ups there is no effect found [71], [72] Using the standardized OTU abundance table we calculated the Jaccard and the ThetaYC dissimilarity indices to identify if our samples had similar community membership and structure. Both indices indicated a strong separation between the 0–4 and 40 cm samples when used to generate UPGMA dendrograms (Jaccard/ThetaYC: parsimony-test: p<0.001) (Figure S1 in File S4). In addition, we find that the 40 cm samples of pockmark and reference sediments are clearly separated (Jaccard/ThetaYC: parsimony-test; p<0.001). The 0–4 cm samples of the reference and pockmark sediments were only significantly different for the Jaccard index (parsimony test; p = 0.049), but not for the ThetaYC index (parsimony test; p = 0.058).

Analysis of similarity (ANOSIM) is a non-parametric test that uses distances based on diversity indices to test for significant difference between samples of multiple groups [73]. Here ANOSIM showed a significant difference between the 0–4 and 40 cm samples for both the Jaccard and the Theta YC indices (R = 0.98; p<0.001). The pockmarks and reference samples of 0–4 cm, were the least divergent in the ANOSIM test (both indices: R = 0,61; p<0.001) while the 40 cm samples show a slightly stronger differentiation (both indices: R = 0.70, p<0.001).

**Figure 4:**
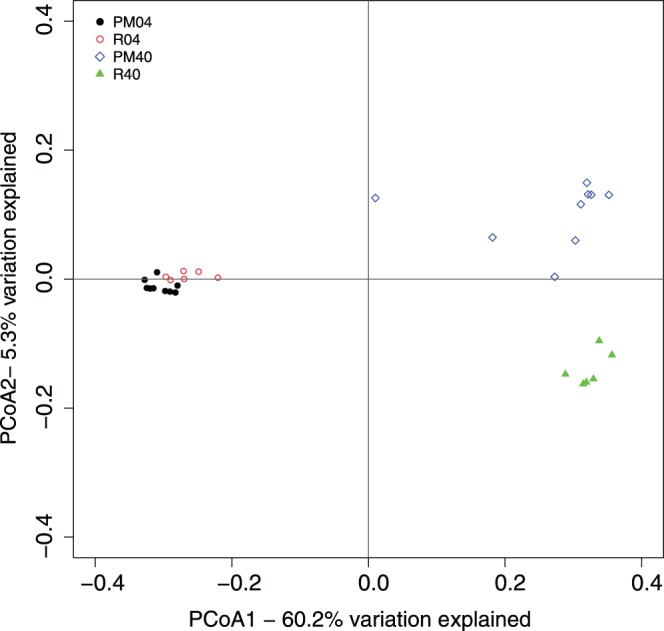
Principal Coordinates Analysis ordination using weighted Unifrac distances. The amount of variation explained for each axis is indicated in percentages. Samples are grouped color wise based on location (pockmark vs. reference sediments) and depth (4 cm vs. 40 cm) in the figure.

Another way of testing community diversity is to use phylogenetics and measure the distances between the samples with the unifrac method [74]. The Unifrac method can be used to analyze the community membership (unweighted) and structure (weighted). Principal Coordinates Analysis (PCoA) of (un-) weighted unifrac distances confirmed the above findings, showing a difference between the reference and pockmark samples in the community structure at depth but not at the surface (Figure 4, Figure S3 in File S4). The axes of the PCoA of the unweighted unifrac distances explained 20.4% and 4.6% of the variation between the communities (data not shown), while the PCoA using the weighted unifrac distances had axes explaining 60.2 and 5.3% of the variation between communities. This indicates that the 40 cm pockmark samples are different from the reference 40 cm samples in regard to community membership and structure.

**Figure 5:**
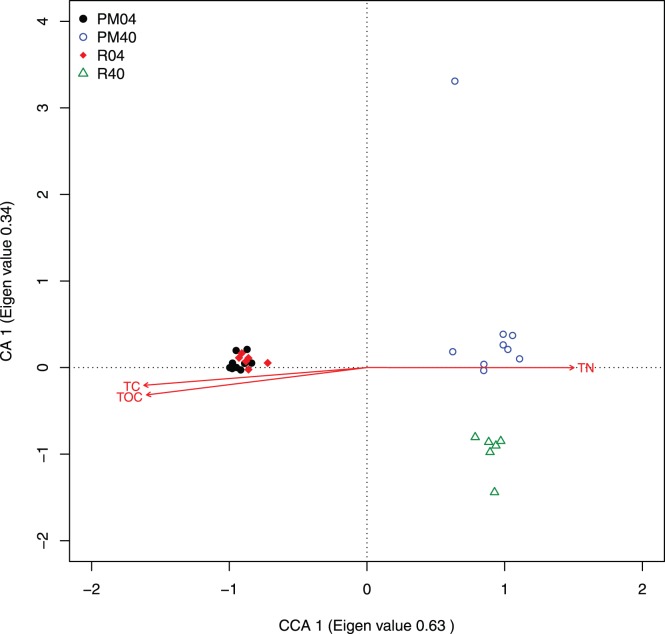
Relationships between bacterial communities of Oslofjord sediments using constrained correspondence analysis. Two-dimensional CCA ordination of the samples using one constrained axis (CCA 1) and an unconstrained axis (CA 1). The constraining factor was Total Carbon. Eigenvalue for both axes are indicated beside each axis. Environmental parameters that significantly (p<0.01) correlated with the ordination were fitted using the envfit command (Vegan package). Abbreviations: total nitrogen (TN), total carbon (TC), total organic carbon (TOC).

To understand the link between the measured chemical parameters and the observed variation in the bacterial community structure we applied constrained correspondence analysis (CCA). CCA confirmed the strong separation of the communities by depth. Further, it indicated a tight grouping of the 0–4 cm communities independent of the location, while the 40 cm communities were clearly separated between pockmark samples and the reference samples (Figure 5). The strongest chemical determinant between the communities was TC, which explained about 11% of the variation between the communities. Additional factors that explain more of the community variation are TOC and TN (Envfit permutation test: p = 0.001). None of the other measured chemical factors were indicated to have a significant influence on the community compositions.

### Metastats Analysis

Beta-diversity analysis showed that at 40 cm depth, community structure differed when comparing pockmark and reference sediments. Using Metastats it is possible to determine which OTUs are significantly different between the various groups [66]. Metastats first normalizes the abundances in the groups before performing a t-test permutation test. Analysis of all the OTUs with a total mean read abundance in one of the groups higher that 0.001 (calculated by Metastats) showed that 58 OTUs had a significant different read abundance between the reference and pockmark samples at 40 cm depth (q-value <0.01) (Table 4). 25 of these OTUs had a higher abundance in the reference samples, while 33 OTUs had more reads assigned in the pockmark samples.

**Table 4:** Metastats results of OTUs with significantly different abundances between the 40 cm pockmark and reference sediment samples.

OTU-ID	SILVA V108 Phylum Classification	SILVA V108 OrderClassification*	Normal 40 cm (abundance %)	Pockmark 40 cm (abundance %)	Metastats q-value (<0.01)
Otu00002	Deltaproteobacteria	Syntrophobacterales	0.090	0.043	0.000
Otu00005	Deltaproteobacteria	Desulfobacterales	0.034	0.025	0.002
Otu00006	Deltaproteobacteria	Desulfobacterales	0.010	0.037	0.000
**Otu00015**	**Gammaproteobacteria**	**Xanthomonadales**	**0.001**	**0.002**	**0.005**
**Otu00018**	**Deltaproteobacteria**	**Desulfobacterales**	**0.002**	**0.007**	**0.003**
**Otu00026**	**Nitrospira**	**Nitrospirales**	**0.016**	**0.006**	**0.008**
Otu00036	Deltaproteobacteria	Desulfarculales	0.010	0.006	0.007
Otu00044	Deltaproteobacteria	Desulfobacterales	0.011	0.004	0.000
**Otu00046**	**Deltaproteobacteria**	**Desulfobacterales**	**0.003**	**0.007**	**0.000**
**Otu00048**	**Acidobacteria**		**0.000**	**0.001**	**0.000**
**Otu00053**	**Deltaproteobacteria**	**Desulfarculales**	**0.004**	**0.006**	**0.008**
Otu00057	Deltaproteobacteria	Desulfarculales	0.007	0.004	0.000
**Otu00062**	**Deltaproteobacteria**	**env.samples** #	**0.001**	**0.007**	**0.000**
**Otu00065**	**Gammaproteobacteria**	**Xanthomonadales**	**0.000**	**0.002**	**0.006**
**Otu00066**	**Gammaproteobacteria**	**Thiotrichales**	**0.000**	**0.002**	**0.001**
**Otu00071**	**Spirochaetes**	**Spirochaetales**	**0.002**	**0.005**	**0.005**
**Otu00072**	**Acidobacteria**		**0.001**	**0.003**	**0.001**
Otu00073	Deltaproteobacteria	Desulfarculales	0.006	0.003	0.003
**Otu00075**	**Deltaproteobacteria**	**Desulfobacterales**	**0.000**	**0.006**	**0.000**
**Otu00090**	**Gammaproteobacteria**	**env.samples**	**0.000**	**0.005**	**0.004**
**Otu00101**	**Actinobacteria**		**0.000**	**0.004**	**0.007**
**Otu00123**	**Spirochaetes**	**Spirochaetales**	**0.001**	**0.003**	**0.001**
Otu00126	Spirochaetes	Spirochaetales	0.003	0.002	0.007
Otu00130	Deltaproteobacteria		0.003	0.002	0.007
**Otu00139**	**Acidobacteria**		**0.000**	**0.002**	**0.004**
**Otu00140**	**Gammaproteobacteria**		**0.000**	**0.002**	**0.000**
Otu00152	Deltaproteobacteria	Desulfobacterales	0.004	0.001	0.000
**Otu00166**	**Fusobacteria**		**0.000**	**0.002**	**0.002**
Otu00169	Nitrospira	Nitrospirales	0.004	0.001	0.000
**Otu00174**	**Actinobacteria**	**Solirubrobacterales**	**0.001**	**0.002**	**0.009**
Otu00179	Gemmatimonadetes	env.samples	0.003	0.001	0.004
**Otu00191**	**Deltaproteobacteria**	**Desulfarculales**	**0.000**	**0.002**	**0.007**
**Otu00200**	**Acidobacteria**	**env.samples**	**0.000**	**0.002**	**0.001**
**Otu00204**	**Deltaproteobacteria**		**0.000**	**0.001**	**0.007**
Otu00208	Deferibacteres	Deferribacterales	0.002	0.001	0.004
Otu00213	Chlorobi		0.003	0.001	0.000
**Otu00238**	**Not assigned**		**0.000**	**0.002**	**0.009**
**Otu00241**	**Gammaproteobacteria**		**0.000**	**0.001**	**0.005**
Otu00253	Gemmatimonadetes	Gemmatimonadales	0.002	0.001	0.001
**Otu00256**	**Deferibacteres**	**Deferribacterales**	**0.001**	**0.001**	**0.005**
**Otu00272**	**Not assigned**		**0.000**	**0.001**	**0.000**
**Otu00282**	**Acidobacteria**		**0.000**	**0.001**	**0.002**
**Otu00290**	**Bacteria**	**env.samples**	**0.000**	**0.001**	**0.007**
Otu00296	Deltaproteobacteria	Desulfarculales	0.002	0.000	0.003
**Otu00297**	**Nitrospira**	**Nitrospirales**	**0.000**	**0.001**	**0.003**
**Otu00322**	**Bacteria**	**env.samples**	**0.000**	**0.001**	**0.000**
**Otu00334**	**Deltaproteobacteria**	**Syntrophobacterales**	**0.000**	**0.001**	**0.001**
**Otu00345**	**Planctomyceters**	**Phycisphaerales**	**0.000**	**0.001**	**0.001**
Otu00353	Fibrobacteres	Fibrobacterales	0.002	0.000	0.004
**Otu00407**	**Spirochaetes**	**Spirochaetales**	**0.000**	**0.001**	**0.006**
Otu00417	Deltaproteobacteria	Syntrophobacterales	0.002	0.000	0.000
Otu00418	Deferibacteres	Deferribacterales	0.001	0.000	0.009
Otu00436	Not assigned		0.002	0.000	0.000
Otu00558	Deferibacteres	Deferribacterales	0.001	0.000	0.000
Otu00607	Deltaproteobacteria	Desulfarculales	0.001	0.000	0.005
Otu00621	Not assigned		0.002	0.000	0.000
Otu00644	Bacteria	env.samples	0.001	0.000	0.001
Otu00802	Deltaproteobacteria	Desulfarculales	0.001	0.000	0.000

Minimum relative abundance as calculated by Metastats >0.001. Text in bold indicates OTUs overrepresented in pockmarks. Classifications are at the order level or higher taxonomical levels.

* Order level classification indicated when identified.

# Abbreviation: env.samples : environmental samples.

### Classification of OTUs

**Figure 6:**
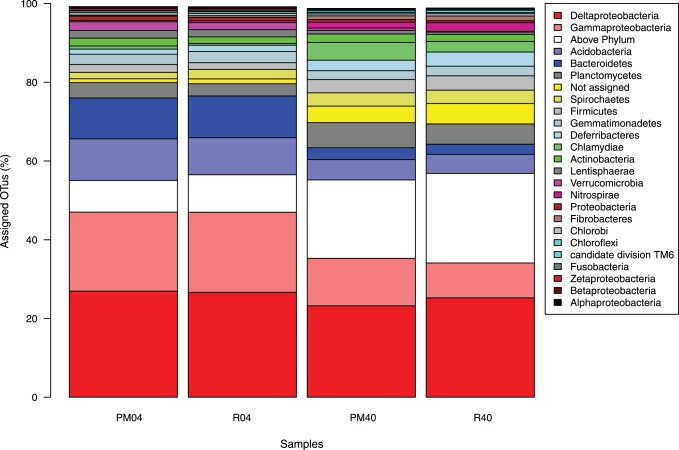
Phylum level abundances of representative OTU sequences. The Lowest common ancestor algorithm was used to classify OTU sequences with blastN against the SILVA V108 SSURef database. The phylum Proteobacteria was split to accommodate for the different abundances within the various sub clades. OTUs that did not classify to the proteobacterial subclades were assigned to the taxon Proteobacteria. The group “Not assigned” consists of sequences with significant blast hits but could not be classified using the set LCA parameters. The group “Above phylum” contains OTU sequences assigned to either the kingdom Bacteria or to cellular organisms. Note that only the top 25 taxa are indicated for clarity.

Taxonomic classification of the OTUs (97% cutoff) against the non-redundant SILVA V108 database at the phylum level shows that a fraction of the OTUs were not assigned (≈0.9–5.2% of total OTUs), did not generate any significant hits (0.01%–0.16%), or were not classified at the phylum level (8%–22%) (Figure 6). For all three groups the 40 cm samples contained a larger fraction of unclassified or poorly classified OTUs. *Proteobacteria* assigned OTUs dominated in all samples (35–49%) but with a higher abundance in the surface samples. Of all Proteobacterial-assigned OTUs most of them are classified as *Deltaproteobacteria* and *Gammaproteobacteria.* Other groups with a higher relative abundance (>2%) of assigned OTUs in the surface than the deep samples are *Acidobacteria, Bacteriodetes*, *Gemmatimonadetes and Verrucomicrobia*. For the 40 cm samples the relative abundances (>2%) of assigned OTUs are higher for *Planctomycetes*, *Spirochaetes, Firmicutes*, *Deferribacteres, Chlamydiae, Actinobacteria* and the phylum *Nitrospirae* (Figure 6).

Taking into account read abundances of the assigned OTU changes the picture of the diversity only slightly (Figure S2 in File S4). Reads assigned to *Deltaproteobacteria* made up almost 40% (+/−3.67%) on average in all samples. The amount of reads in the non-assigned pool consisted of a smaller fraction of the complete dataset suggesting that many reads belonged to low abundance OTUs. Interestingly, the amount of reads assigned to *Nitrospirae* represented a much larger fraction of the diversity, especially for the R40 samples (6.0% +/−3.3%).

Classifications of the OTUs identified with the Metastats analysis gave an extra angle on the differences between the PM40 and R40 samples indicating that 4 OTUs could not be reliably classified against the SILVA V108 database. Of the unclassified OTUs, two were more abundant in the PM40 samples while the other two had higher abundances in the N40 samples. The other 54 OTUs were assigned to various taxonomical levels (Table 4), with most of the OTUs assigned to Delta- or Gammaproteobacterial orders such as the *Desulfarculales*, *Desulfobacterales*, *Thiotrichales* and the *Xanthomonodales*. The latter two taxa had higher abundances in the 40 cm pockmark samples, while the former two taxa showed a mixed pattern with OTUs having higher abundances in either 40 cm pockmark or reference samples. A similar pattern was also found for OTUs in the orders *Deferribacterales, Nitrospirales, Spirochaetales and the Syntrophobacterales*. For the taxa *Acidobacteria*, *Actinobacteria*, *Fusobacteria* and *Phycisphaerales* the OTUs had higher abundances in the PM40 samples. In contrast, OTUs belonging to the orders *Chlorobi*, *Fibrobacterales*, *Gemmatimonodales* had higher abundances in the R40 samples.

## Discussion

Soils and sediments are by far the most diverse microbial ecosystems present on this planet. A recent analysis by the International Census of Marine Microbes (ICoMM) found almost 60000 OTUs in 72 coastal benthic samples with almost 50% of the OTUs only found as singletons [75]. This suggests that sediments show a high microbial diversity per site but also between sites. Thus, it is conceivable that the microbial community diversity of inactive pockmark sediments would be different from normal coastal sediments. Nonetheless, the microbial community composition of inactive pockmark sediments may potentially be influenced by occasional seepage of freshwater, by different organic matter deposition rates, or by a different bioturbating macrofaunal community composition as compared to the surrounding sediments [9], [11], [14], [22]. These influencing factors could be due to lower porewater salinity, or different carbon and/or nitrogen concentrations in the pockmark sediments as compared to the surrounding sediments.

### Pockmark Sediment Chemistry

To quantify the influence of freshwater seepage we can measure Na^+^ or Cl^−^ concentrations that determine for a large part the salinity of the sediments. It is well established that salinity gradients affect microbial community composition in marine and freshwater sediments, suggesting that if inactive pockmarks experience occasional freshwater seepage it could affect the pockmark sediment communities [76], [77]. The chemical analysis of the Oslofjord sediments did not indicate any differences in Na^+^ or Cl^−^ concentrations between 0–4 and 40 cm sediments inside our outside the pockmarks, suggesting that at these depths freshwater seepage has no influence on the sediment communities.

Bioturbation or different organic matter deposition rates, which both are implicated in affecting the microbial community composition in pockmark sediments, may alter the biogeochemistry of the sediments due to differences in the carbon and nitrogen concentrations. A comparison of the chemistry concentrations between the pockmark and reference sites for both 0–4 and 40 cm showed significant differences for TN, TC, TOC and PAH concentrations between the two depths of the sediments. At 40 cm depth the TN concentration is higher than at the surface of the sediments. The TN in our samples is mostly likely composed of the NH_4_^+^ and NO_3_^−^ ions as in other organic rich sediments [78], [79]. The composition of TN in such sediments changes with NO_3_^−^ concentrations decreasing and NH_4_^+^ concentrations increasing with depth due to nitrate reduction coupled to organic matter degradation [78]–[80].

In contrast to the TN concentrations, TC, TOC and PAH levels were highest at 0–4 cm and much lower at 40 cm depth suggesting that carbon sources are deposited from the water column. In addition, the PAH ratios calculated here all seem to indicate that combustion of wood is the major source of the PAHs (ANT178>0.1; BAA228>0.2), which is an additional sign of deposition of carbon from the water column [38].

Surprisingly, the concentrations of NPOC measured from the porewater – basically dissolved organic carbon (DOC) – did not show a depth-related difference. TC and TOC were measured from ground and dried sediments and in theory would be based on dissolved and sediment adhered organic carbon. The amount of DOC detected in porewater is dependent on the sorption coefficient of water soluble organic carbon attached to the sediment [81]. In the literature we find several explanations for the DOC concentrations at different sediment depths [77], [78]. A large fraction of the organic matter is adsorbed to the surface of sediment particles during sedimentation, and only a small fraction will be released again into the pore water and detected as DOC [82]. Alternatively, with depth the potential of the microbial community to hydrolyze DOC decreases allowing for a persistence of DOC concentrations with depth [83]. Our results suggest that TC/TOC concentrations decrease with depth, while NPOC concentrations remain fairly constant, but this remains speculative since we did not measure the intermediate depths. Nonetheless, TOC concentrations found in another study from the Oslofjord did decline with depth, however only after an initial increase at the top sediments [84]. Nonetheless, we find similar values for the surface as well as the deeper sediments in our study as in Arp et al., (2011) suggesting the same concentration pattern in our sediments.

Comparison of the chemical concentrations at the same depth between the pockmark and reference sediments did not show significant differences for the 0–4 cm samples. For the 40 cm depth a small and significant difference was found for TC, TOC and NPOC between the pockmark and the reference sites. Interestingly, TOC and NPOC concentrations showed opposing patterns with TOC concentrations being lower in the pockmarks while NPOC showed higher concentrations in the pockmarks (Table 2). These contrasting results could be caused by differences in the release or hydrolysis of DOC within the sediments of our study sites [83]. It also suggests that between pockmark and normal coastal sediments degradation rates of organic matter might be different.

The biogeochemistry analysis of the pockmark and reference sediments does not distinguish between the two factors, differences in either hydrodynamic regime or bioturbation, as a source for microbial community differences. As we did not determine the macrofaunal community composition in each of our samples sites we cannot conclude concerning the importance of this factor for the chemical differences. Regarding different hydrodynamic regimes within and outside the inactive pockmarks, it is known that different deposition rates of organic matter can influence the microbial community richness, but so far this has only been shown in oligotrophic marine surface (0–5 cm) sediments [33]. The Oslofjord is not an oligotrophic environment, and in line with Bienhold et al., (2012) a difference between the 0–4 cm microbial communities of the pockmarks and the references sediments was not found, which indicates that organic matter deposition rates are similar at both sites [84]. An additional factor influenced by different sedimentation rates could be the grain size of the pockmark sediments [14]. Interestingly, several recent studies identified grain size as an environmental variable that influences bacterial community composition in sediments and is closely correlated with TOC concentrations [85]–[89]. Basically, with increasing mud content of sediments TOC concentrations increase as well as total microbial and bacterial biomass [89]. Although highly speculative, this suggests that the pockmark in our study have a slightly larger grain size as well as slightly lower bacterial abundances. Regardless, our chemical analysis indicates that at 40 cm depth there is a significant difference between pockmarks and surroundings sediments and this is supported by our microbial community analysis.

### Effect of Singleton Removal on Alpha Diversity

454 pyrosequencing of the V3 region of the 16S rRNA revealed a tremendous diversity in our sediment samples. Our results indicated that the communities are extremely diverse with more than 20000 OTUs found in 30 samples at a 97% similarity cut-off (Figure 2). Almost 50% of the detected taxa were represented by a single sequence as found in other studies [75]. It is however unclear if all these single sequences represent a real rare biosphere species or are due to PCR or sequencing errors [68]. Removal of the unique sequences reduced the total diversity of this study to 9246 OTUs with a significant reduction of observed OTUs per sample (Figure 2, Table 3). This did not alter the significant difference between the rarefaction curves of the 40 cm and the 0–4 cm samples with the former indicating a higher general diversity (Figure 2, File S3). In contrast, for most alpha diversity estimators, except the non-parametric Shannon index, removal of singletons did eradicate the significant difference in observed diversity between the two studied depths (File S3). The non-parametric Shannon index, which measures both richness and evenness indicated that there is a significant differences between the 0–4 and 40 cm communities, suggesting that the species are more equally distributed deeper in the sediments than at the surface with rare species more easily detected [33], [90], [91]. In line with this we find not only more OTUs, but also more singleton OTUs per sample at 40 cm depth (Table 3).

The removal of singletons improved classification of the OTUs considerably. With singletons included we found 16–27% of the OTUs unclassified (data not shown), while singleton removal reduced the number of unclassified OTUs to 0.9–5.2%. This implies that most of the removed singletons contained artifacts not representing unidentified species [67]–[70].

Our results with singletons removed still indicate the presence of an extensive rare biosphere in the sediments accounting for most of the diversity. The high diversity of the samples is also evident from the amount of OTUs shared between the four groups where we only found 28 shared OTUs between all samples (Figure 3). Within each group the amount of shared OTUs is considerably higher but is still approximately 10% of the total OTUs detected per sample. This suggests that our sequencing effort should have been larger to increase the overlap between the communities [92]. Even so, compared with relevant studies we find similar levels of diversity in the Oslofjord sediments [44], [93].

### Pockmark Sediment Community

To address if there is a difference between the pockmark and control sediment communities, we used beta-diversity indices based on either sequence similarity (OTUs) or phylogenetic distances. In the latter case we used (un-) weighted Unifrac to measure similarity between the communities [94], while in the former case we used OTU community composition to either calculate the Jaccard, ThetaYC or Chi-square (CCA analysis) distances.

Both Unifrac and the OTU based analyses indicated a significant difference between the 0–4 cm and 40 cm communities and between the PM40 and R40 samples. At the 0–4 cm depth only the Jaccard index showed a significant difference between pockmark and reference sediments, but this was not identified with ANOSIM. The significant difference between the 0–4 and 40 cm communities was expected and is in line with previous studies and our alpha diversity analysis. It is well known that microbial community composition changes with depth following the nutrient status and redox potential within sediments [52], [88], [95].

The difference between the 40 cm communities of the pockmarks and the reference samples is not easily explained. We therefore used CCA to correlate the chemistry data with the OTU abundances of each of the communities (Figure 5). In line with the chemistry results, the CCA indicated that TC, TOC and TN were the main determinants explaining the community differences between the samples, and that they explain mostly the difference between the 0–4 and 40 cm samples. Additionally, the vectors for TC and TOC seem to indicate that the reference samples have slightly higher concentrations for these variables, which could be an explanation for the small differences in community composition between the pockmark and reference samples.

### Taxonomic Diversity

The results from the diversity analysis indicated a significant difference between the 40 cm communities of the pockmarks and the reference sites. An analysis of the phylum level classifications indicated several groups with different OTU abundances with regard to sediment depth. The number of OTUs assigned to only the taxonomical level Bacteria (above phylum), non-assigned and no-hits was higher in the 40 cm samples. This result could be due to erroneous doubletons, or the presence of unidentified phyla in the sediment samples not present in the SILVA V108 database. Recently, several novel phyla were targeted using single-cell genome sequencing suggesting that the diversity of environmental communities is far from completely mapped [96].

The classification results of the OTUs identified at 97% sequence similarity cut-off indicated the dominance of Delta- and Gammaproteobacterial species in the 40 cm sediments of all samples followed by *Acidobacteria*, *Bacteriodetes*, and *Planctomycetes* (Figure 6). These groups are known to dominate marine sediments and the diversity within these groups is comparable to other studies [44], [75], [93].

The Delta- and Gammaproteobacterial spp. contain many sulfate reducers which are typical constituents of marine sediments. Interestingly, the diversity of *Deltaproteobacteria* seems comparable between the PM40 and R40 samples (Figure 6). This is as well reflected by the Deltaproteobacterial OTUs identified with Metastats (Table 4). Their classifications at the order level show that some orders can be found as different OTUs with significantly different abundances in either the PM40 or R40 samples. For the *Gammaproteobacteria* we find OTUs belonging to the *Xanthomonadales* and *Thiotrichales* being overrepresented in the PM40 samples compared to R40, which coincides with a general higher diversity of *Gammaproteobacteria* in the PM40 samples (Table 4, Figure 6, Figure S2 in File S4). The *Thiotrichales* contains species such as the sulfur oxidizing *Beggiatoa* and *Thioplaca* spp. that use anaerobic reduction of nitrate coupled to oxidation of organic matter and/or sulfur [97]. The order *Xanthomonadales* contains important plant pathogens such as *Xylella* spp., but has recently also been shown to contain PAH degraders, which suggest that *Xanthomomodales* in sediments are involved in the breakdown of complex organic matter [98].

At 40 cm depth both the *Acidobacteria* and *Bacteriodetes* OTUs have lower diversity compared to the 0–4 cm samples (Figure 6). Among the *Bacteriodetes* OTUs there were none that were overrepresented between the PM40 or R40 samples, while for *Acidobacteria* we find several OTUs overrepresented in the PM40 samples (Table 4). *Acidobacteria* were first described in highly acidic soils, but their presence in marine and freshwater sediments has been shown repeatedly with OTU abundances around 5% [44], [93], [99]. A recent comparative genomic analysis of three Acidobacterial isolates indicated that this group is capable of degradation of complex compounds such as cellulose, chitin, using nitrate/nitrite reduction, indicative of their importance as organic matter degraders in sediments [100]. Other known degraders of complex compounds are found in the phylum *Bacteroidetes* and their abundance is tightly linked with organic matter concentrations [83], [101].

In contrast to *Gammaproteobacteria*, *Acidobacteria* and *Bacteroidetes*, we find higher diversity levels for the *Planctomycetes* at 40 cm, which coincides with the phylum *Spirochaetes* and higher read abundances for the *Actinobacterial* assigned OTUs (Figure 6, Figure S2 in File S4). Further, these taxa also contain several OTUs that were overrepresented in the PM40 samples. As a taxon *Planctomycetes* is well known for its capacity to perform anaerobic ammonium oxidation (anammox), but has been implicated in the degradation of organic matter in the environment as well [86], [102], [103]. The single OTU overrepresented in the *Planctomycetes* belongs to the order *Phycisphaerales* (Table 4). This order is represented by an anaerobic isolate capable of reducing nitrate and breaking down agar [104]. The phylum *Spirochaetes* is characterized by mostly anaerobic bacteria degrading complex carbohydrates and fermentation [105]. Finally, the *Actinobacteria* are well known for their capacity to mineralize organic matter such as cellulose or chitin [106].

Bacterial phyla that have overrepresented OTUs among the R40 samples can be classified as *Nitrospirae*, *Gemmatimonadetes* and the *Deferribacteres* (Table 4). Members of the *Deferribacteres* are often identified in extreme environments such as hydrothermal vents or oil production facilities, but are occasionally found in sediments at low abundances [107], [108]. Members of this phylum use nitrate/sulfur/sulphate reduction to oxidize small carbohydrates such as fumarate, malate and acetate. In line with this anaerobic lifestyle, we find higher *Deferribacteres* diversity at 40 cm compared to 0–4 cm (Figure 6) [109]–[111]. The phylum of *Gemmatimonadetes* is little characterized and mainly found in soils [112]. Fertilization of soils increases the *Gemmatimonadetes* OTU abundances indicating their role as heterotrophic bacteria influenced by nitrogen and carbon [113]. Interestingly, they can be found in the marine sediments such as the oxic sediments of the Pacific abyss and in the Oslofjord sediments analyzed here [107]. Finally, the highest diversity levels for the phylum *Nitrospirae* were found in the R40 samples, with several OTUs overrepresented (Figure 6, Table 4). The *Nitrospirae* are known as chemoautotrophic bacteria involved in the nitrogen cycle and several members are involved in nitrite oxidation while sharing genes with anammox bacteria [114], [115].

Many of the identified overrepresented OTUs belong to bacterial orders that are not well studied, and the metabolic properties derived from the literature are often based on a few well-studied isolates. We find that most of the above mentioned taxa are involved in nitrate, nitrite or sulfur reduction associated with oxidation of organic matter. Interestingly, the molecular complexity of the degraded organic matter is different between the phyla. Most of the overrepresented OTUs in the PM40 sample are implicated in the degradation of complex carbohydrates. In contrast, the overrepresented OTUs in the R40 samples either breakdown small carbohydrates (*Deferribacteres spp.)* or they might have a autotrophic lifestyle (*Nitrospira*e spp.) which does not rely on organic matter breakdown.

All the 40 cm samples had similar TN concentrations, but there was a clear difference for TC, TOC and NPOC concentrations between the pockmark and the reference samples at this depth (Table 2). For TC we can explain the lower concentrations in the PM40 samples by the increased abundance of OTUs related to phyla with the capacity to degrade complex macromolecules that are less prominent in the reference sediments. In addition, the reference samples could have a community more adapted to use small soluble carbohydrates decreasing the DOC concentrations in the porewater. Our findings suggest that slightly different in-situ sediment conditions can alter the C:N ratio of the sediments. Furthermore, this work supports that the surface sediment communities are highly influenced by the above-lying water column, while communities at 40 cm depth are site specific [52], [88], [89].

## Conclusions

We have shown that microbial communities in inactive pockmark sediments are highly diverse and have a depth-related structure just like normal sediment communities. Despite being influenced by the same water body of the Oslofjord, we demonstrate that the pockmarks have a different community structure at 40 cm depth compared to surrounding sediments. In contrast, surface sediments of inactive pockmarks are indistinguishable from the surrounding sediments. Our work gave no indications on which factors cause this depth difference, but there are hints that we should investigate the factors that affect degradation rates of complex carbohydrates. In addition, our findings indicate that we can use inactive pockmark sediments for exploring the influence of physical variables, such as sedimentation rates, grain size and sediment porosity on microbial community structure. Finally, these results have implications for research comparing microbial sediment communities in relation to geological features. The sediment surface communities do not show differences, while deeper buried communities seem to be influenced by local conditions. It suggests that for the deeper sediment layers local conditions are stronger determinants for the microbial community composition and structure than at the sediment surface.

## Supporting Information

File S1 **This file contains Table S1–Table S4.** Table S1, Pairwise distances between sample locations. Table S2, Visual observations for each core taken in the present study. Table S3, Combined chemistry data from the different measurements done on the Oslofjord samples. Table S4, Diversity estimates for bacterial sequences with confidence intervals.
(XLSX)

File S2 **Supplementary information with the sequences of the Primers, MID-tags and adaptors used for amplification of the 16S rRNA V3 region.**
(XLSX)

File S3 **This file contains four tables (Table S1–Table S4) on the comparison of the alpha diversity with and without unique reads included.** Table S1, Comparison of alpha diversity estimators with and without removal of unique sequences. Table S2, Overview of the average differences per alpha diversity estimator. Table S3, Side by side comparisons of alpha diversity estimators. Table S4, Kruskal-Wallis test to test for significance in the Alpha diversity estimators with and without removal of unique sequences.
(XLSX)

File S4 **This file contains Figure S1–Figure S3.** Figure S1, UPGMA dendrograms of the Oslofjord pockmark and reference sites sediment communities. Figure S2, Phylum level abundances of all sequences. Figure S3, Principal coordinates analysis ordination using Unifrac distances.
(DOCX)

File S5 **Supplementary archive file containing the files: final.fasta, final.names, final.groups and final.taxonomy, which were used to do the diversity analysis for this publication.** The tar archive can be uncompressed using uncompression software such as tar or winzip. To uncompress the data (≈30 Mb) with the tar software type at the command line: tar -xzvf additional_file_5_final_data.tar.
(TAR)
