# Supplementary material for: Linking Geology and Microbiology: Inactive Pockmarks Affect Sediment Microbial Community Structure
Source: PLoS One. 2014 Jan 24;9(1):e85990. doi: 10.1371/journal.pone.0085990 (PMC3901666; doi:10.1371/journal.pone.0085990)
Supplement: File S4 — This file contains Figure S1–Figure S3. Figure S1, UPGMA dendrograms of the Oslofjord pockmark and reference sites sediment communities. Figure S2, Phylum level abundances of all sequences. Figure S3, Principal coordinates analysis ordination using Unifrac distances. (DOCX) [file pone.0085990.s004.docx]

**Supporting_information_file 4. Supplementary Figures.**

**Figure legend**

**Figure S1. UPGMA dendrograms of the Oslo Fjord pockmark and reference sites sediment communities.** A) UPGMA dendrogram based on the Jaccard index. B) UPGMA dendrogram based on the ThetaYC index. Dendrograms are shown as unrooted trees. Values indicate bootstrap confidence scores (1000 replicates) above 0.5.

**Figure S2. Phylum level abundances of all sequences.** The Lowest common ancestor algorithm was used to classify sequences with blastN against the SILVA V108 SSURef database. The phylum Proteobacteria was split to accommodate for the different abundances within the various sub clades. The group not assigned consists of sequences with significant blast hits but could not be classified using the set LCA parameters. Note: only the top 25 taxons are indicated.

**Figure S3. Principal coordinates analysis ordination using Unifrac distances.** A) PCoA of unweighted Unifrac distances. B) PCoA of weighted Unifrac distances. The amount of variation explained for each axis is indicated in percentages. Samples are grouped color wise based on location (pockmark vs. reference sediments) and depth (0-4 cm vs 40cm) in the figure.

**Figure S1**

**Figure S2**

**Figure S3**
